# Contributors to COVID-19-Related Childbirth Anxiety among Pregnant Women in Two Pandemic Waves

**DOI:** 10.3390/ijerph20010110

**Published:** 2022-12-22

**Authors:** Orit Taubman–Ben-Ari, Miriam Chasson, Hilit Erel-Brodsky, Salam Abu-Sharkia, Vera Skvirsky, Eran Horowitz

**Affiliations:** 1 The Louis and Gabi Weisfeld School of Social Work, Bar Ilan University, Ramat Gan 5290001, Israel; 2 The Sackler Faculty of Medicine, Tel Aviv University, Tel Aviv 6997801, Israel; 3 Maccabi Healthcare Services, Tel Aviv 6812509, Israel

**Keywords:** COVID-19, pregnancy, childbirth anxiety, distress, optimism, self-compassion

## Abstract

COVID-19 has impacted all levels of daily life for people everywhere, with particularly serious implications for pregnant women. This paper examines the COVID-19-related childbirth anxiety (CCA) of Israeli women in the first two waves of the pandemic. We first present two psychotherapeutic case studies with pregnant women in the two waves. This is followed by an empirical study that compared the contribution of background variables, psychological distress, economic concerns, and personal resources to CCA in two samples, Wave 1, March–April 2020 (*n* = 403) and Wave 2, September–October 2020 (*n* = 1401), and two subpopulations, Jewish and Arab women. Findings reveal that CCA was significantly lower in Wave 2 than in Wave 1. Furthermore, poorer health, higher education, being an Arab, later gestational week, at-risk pregnancy, wave, higher psychological distress, greater economic concerns, and lower self-compassion contributed to higher childbirth anxiety. Wave moderated the association between optimism and anxiety. The findings of the empirical study, together with insights from the case studies, provide evidence of a decrease in CCA later in the crisis, and indicate the significance of resources for coping with the psychological implications of the pandemic. Moreover, they suggest the importance of empowering self-reliance techniques, such as self-compassion, which was significantly associated with lower anxiety, above and beyond the background and psychological variables. Clinical Impact Statement: Using both psychotherapeutic cases and empirical findings, this study points to the risk and resilience factors that contributed to pregnant women’s COVID-19-related childbirth anxiety (CCA) in the first two waves of the pandemic. The study suggests that CCA was higher in the first wave, as well as among women from a minority group. At the same time, the research shows that resilience resources of optimism and self-compassion contributed to the reduction of anxiety. These findings may guide interventions for the vulnerable group of pregnant women in times of crisis.

## 1. Introduction

The outbreak of COVID-19 impacted every level of daily life [1,2], with the overwhelming uncertainty regarding countless aspects of both the near and more distant future leading to traumatic changes in the lives of people around the globe, as well as high levels of distress, depression and anxiety [3,4]. Among those most seriously affected by the crisis were pregnant women due to give birth in the near future [5,6,7]. In addition to their usual anxieties, these women were troubled by virus specific concerns, such as being infected, the welfare of the fetus, not getting the necessary prenatal care, and the need to visit community clinics and physicians. Indeed, at the outbreak of the pandemic, people were advised to stay home and communicate remotely with their physicians [5,8]. Moreover, a systematic review of the psychological impact of infectious disease outbreaks on pregnant women highlighted the importance of disrupted expectations of birth [9].

Despite their concerns, pregnant women knew they would eventually have to go to the hospital to deliver their baby, that is, they would have to spend time in a public place, where the chance of infection was greater, most likely increasing their anxiety and adding to the common fear of childbirth (FOC) [10]. Indeed, a study conducted in Italy during the first lockdown indicated that whereas prior to the crisis childbirth expectations were characterized by joy, safety, and serenity, the dominant responses after the outbreak of the pandemic were loneliness, anxiety, danger, and worry [11]. FOC and prenatal stress have been found to have negative consequences for the woman’s physical and mental health, as well as to be associated with higher rates of adverse birth outcomes [10,12,13].

The literature indicates that both internal resources (e.g., poor mental health, anxiety disorders, previous trauma) and external resources (e.g., lack of social support, economic problems [14]) play a role in FOC. Moreover, the internal resource of self-compassion was found to serve as a shield against anxiety in the early stage of the pandemic [6], and was associated specifically with a lower level of COVID-19-related childbirth anxiety (CCA). However, although the external resource of perceived social support has been shown to be a major source of protection against stress and anxiety [15,16], it was not found to contribute to lower CCA. The authors suggest that the protective role of social support may have been diminished by the need for social distancing and the woman’s awareness that her support system would not be available to her when she gave birth.

The present paper spotlights pregnant women’s psychological state in the first two waves of the pandemic, combining two psychotherapeutic case studies with an empirical study that examined the contribution of background variables and personal resources to CCA. The investigation compared samples from two waves of the pandemic in Israel, Wave 1 in March–April 2020, and Wave 2, six months later, in September–October 2020, and examined the possibility that research phase moderated the associations between the other variables and CCA. It was assumed that while women might have adjusted to the crisis during the six months since the outbreak of the pandemic, the second wave was a much more difficult experience, with many more confirmed cases of COVID-19 and about ten times as many mortalities, so that people may also have been more fearful, exhausted and frustrated [17].

As the stress of preparing for birth during a pandemic may elevate women’s risk of experiencing anxiety, over and above sociodemographic, obstetrical, and other health factors [18], we considered the role in the two waves of three personal resources that might help in coping with the added stress. The first is **self-compassion** [19], which refers to self-kindness (the ability to be warm and understanding toward ourselves), common humanity (the recognition that vulnerability and imperfection are part of our shared experience), and mindfulness (the ability to be open and receptive to our own thoughts and feelings, whether positive and negative). Studies indicate that self-compassion aids in coping more adaptively with stress in general [20], has been associated with lower depression and anxiety among pregnant women during the perinatal period [21,22], and lower COVID-19-related childbirth anxiety during the pandemic [6].

Another relevant personal resource is **optimism**, defined as an individual’s positive perception and expectations for the future [23]. Empirical evidence indicates that optimism is associated with positive outcomes (e.g., better mental and physical health), both in the general population [23] and among pregnant women [24], as well as during the pandemic [25], and is negatively associated with FOC [26].

Finally, we examined the role of **perceived social support**, which relates to the belief that a person is loved, valued, and part of a social network of mutual ties and commitment [27]. As noted above, social support has been found to function as a protective factor against distress during pregnancy in general [28], and during the pandemic [8] in particular, as well as to contribute to reduced FOC [15,16].

### The Current Study

The uncertainty and threat accompanying the current crisis are likely to increase the stress associated with childbirth as they place additional burdens on women due to give birth during the pandemic. To the best of our knowledge, the few studies which relate to this issue were conducted in the early days of the pandemic. The current study sought to understand the factors that contribute to pregnant women’s anxiety over giving birth in the shadow of COVID-19 during the second wave of the pandemic in Israel as well.

Below we present two clinical cases conducted by the third author, a psychoanalyst and psychoanalytic psychotherapist. This is followed by a description of the empirical study. Finally, we attempt to combine the insights gained from the two methodologies into a coherent understanding of pregnant women’s mental state during the first two waves of the pandemic.

The following case studies demonstrate the differences in the experience of two women who were pregnant during the COVID-19 pandemic, in the first wave and in the second one. The first lockdown was strictly enforced and the anxiety and uncertainty among the entire population was high relatively to the second wave. Both patients were anxious women, who had little self-compassion, were shaken easily, and with a tendency towards a pessimistic view of reality. Yet, it was evident that the patient that was pregnant in the first wave felt a paralyzing anxiety, a nameless dread, surrounding the pregnancy and birth, felt that her resources were greatly depleted, experienced social distance and helplessness from her caregivers, her relatives and her doctors. In contrast, the second patient, who was pregnant in the second wave, experienced lower levels of anxiety, felt that she was able to manage her fears and anxieties, that her therapy could hold and contain her, and trusted both her own abilities and her baby’s ability to survive the pregnancy during the pandemic, and towards the expected childbirth.

## 2. Part 1: Clinical Case Studies

For pregnant women across the world, the first wave of COVID-19 upped the stakes of maternal responsibility and introduced (or heightened) another element of their experience: fear [29]. Maternal self-focus became more critical, and the weight of responsibility increased significantly. The pregnant woman had to be entirely self-focused in order to protect the *other* within, a “not yet” subject whose only ties to the person it inhabits are ones of biology, responsibility, and the promise of a future relationship [30].

Concern for the well-being of the fetus, together with overall uncertainty, peaked during the first wave of the pandemic. In addition, the family and friends of pregnant women were also dealing with their own fears and were therefore less available to provide support. In fact, they often fueled the fears and guilt of a pregnant women over endangering herself and her fetus by leaving the house. Indeed, the first lockdown was traumatic as it created a kind of a caesural partition between the past and the present [30].

However, Dodds [31] claims that as more information was gathered regarding the pandemic, the population as a whole, and pregnant women in particular, were able to regain their ability to think. Hence, during the second wave it became clear that pregnant women were more optimistic about the possibility of a new vaccine that would bring an end to the pandemic, and had more information regarding the situation in hospitals. By the same token, by that point, their support networks were also able to function more efficiently.

### 2.1. Case Study 1: First Wave of the Pandemic

N. started analysis many years prior to her pregnancy. She was a highly anxious and insecure young woman struggling to separate from her parents. At a certain point during analysis, she left her parents’ home and travelled to the Far East for several months. Upon returning to Israel, she began pursuing academic studies.

Aggression, anxiety, and a problem in delaying her need for immediate gratification were the main focus of therapy at that time. Our relationship was close and meaningful, but all the while there was constant turbulent transference. She often assumed the role of her parents, whom she experienced as invasive and ruthless. I, in turn, became either the insecure daughter or, often, the mercilessly assaulting mother/analyst seeking to end the pain that emerged within our relationship. During this period she experienced every interpretation I proposed as a command, and I had to treat her with extreme gentleness. At the same time I had to interpret the mutual pain that stemmed from her inability to receive my nourishment; for her, nourishment meant invasiveness. I also had to endure my own aggression toward her when I felt like a potentially violent mother.

Our ability to “survive” this complex period assisted her progress in life. She succeeded professionally, and married two years prior to the pandemic. Although her marriage was fraught with anxiety and doubts, including the issues of invasiveness and dominance, there was also open communication and friendship. N. became pregnant just before the first lockdown, when the pandemic was still inconceivable. A few weeks later, reality changed completely. During the lockdown, we began holding our sessions on Zoom. She avoided leaving the house, was anxious about her fetus, and was fearful of catching the virus and endangering both her fetus and herself. Her anxiety escalated when a problem was detected during one of her prenatal check-ups which led to a series of additional tests. She panicked and sought assistance from doctors specializing in genetics. She felt alone, describing her husband as “indifferent” to her emotional state, since he was mainly anxious about their finances. Furthermore, her parents did not visit or offer help owing to their fear of the virus. She also felt that the healthcare system was unable to meet her needs. In analysis, she was angry, demanding, and frightened, and I found it difficult to calm and comfort her, since I, too, was frightened.

In one of our sessions she remained silent for some time. Finally, she recalled a dream: “I was running about on the road with a baby in a stroller. He was screaming and I couldn’t calm him down. We were walking and cars were going by quickly. I didn’t understand how I got there, and then the stroller disappeared, and I didn’t notice. I just kept running, looking for someone to save me. After a while I realized that I had left the baby behind. How could I forget him? How could I leave him to die? I ran all over looking for the stroller. Suddenly, the highway was empty, totally empty. No cars at all. I was running, screaming, and then I heard a painful cry. I think it was my baby. I woke up crying and frightened. What kind of a mother would I be if I forgot my baby like that in the middle of a highway?”

Me: “At this point during the pandemic, everything is running, changing, and also stopping in an abrupt and terrifying way. I think that we’ve all become helpless, frightened parents without any answers, and like babies we’re scared and crying. You were unable to find your baby without me hearing your painful cry, without us soothing the baby who is you”.

N. cries: “I wish someone would say that everything’s alright…that everything’s okay. I’m so worried about him. I’m afraid my dream of losing him like that means I’ll be a neglectful mother”.

Me: “I think your emerging motherhood is very positive, that despite your painful cry, you’re looking for your baby even now, even amidst the terror you feel”.

After this conversation, N. was able to share her fears with her husband. He, in turn, was able to support her emotionally despite his own worries. Her anxiety about the well-being of both her fetus and herself continued throughout her pregnancy in the shadow of the pandemic. Thankfully, the baby was born healthy.

N. expressed guilt and fear about her inability to protect her fetus. Her dream depicted an image of trauma with empty roads, symbolic of the first lockdown. Furthermore, the empty roads may symbolize the difficulty of thinking clearly and the inability to emotionally extricate oneself from the sense of overwhelming fear. Those close to her found it hard to assist and soothe her, and it even took me considerable time to retrieve my ability to think and dream, and help her envision a healthy pregnancy and a positive future.

### 2.2. Case Study 2: Second Wave of the Pandemic

S. had been in therapy for four years. She was temperamental, anxious, and pessimistic, and was often envious. She felt excluded and scorned by others, while she herself degraded and humiliated them. She had a love-hate relationship with her mother, and our relationship was also rocky. During the first lockdown she asked that we meet twice a week, instead of once, and demanded that the second session be free of charge. The topic of payment led to a meaningful discussion about feelings of envy and the difficulty of depending on others. Engaging more intensively in analysis soothed her. During that time, she became pregnant. Although the pregnancy was accompanied by concerns of infection and the health of her fetus. she was nonetheless hopeful that a vaccine would be invented and that would alleviate her anxiety.

During the second lockdown, I offered my patients the choice of meeting either on Zoom or in my clinic. S. chose Zoom. However, when I later returned to my clinic, she observed that the background had changed. She became more aloof and seemed hurt that I had “continued on without her”, assuming that I did not miss her and that I preferred she not come to the clinic for fear she would infect me. She was quiet and appeared lifeless. She told me that she saw her neighbor, who was not pregnant, leaving for work every day and she wondered whether she should return to work or continue working from home. I told her that, like her neighbor, I appeared to have moved on without her and that she wanted to be reassured that I missed her too, felt her absence, and wanted her to return. For our next session, she came to the clinic and said that after our last conversation, she had imagined a dove with an olive leaf, like the one on Noah’s Ark after the flood.

Me: “Like the dove, you, too, found the strength to fly and come here today, you found hope that the waves would grow calm and you’d be able to peacefully gather leaves to build a nest for your baby”.

S. fell silent and cried for a long time. Then she laughed: “Today I feel more like a turkey than a dove, but still, I’m here and I come in peace”.

S.’s therapy during the second wave of the pandemic focused on envy and exclusion, but not terror. She expressed hope (an olive leaf) and was able to imagine that the pandemic (flood) would be over in the near future and she would feel calm again. The sense of guilt regarding the risk to her fetus surfaced to a lesser degree, and we were both able to maintain our ability to think.

### 2.3. Clinical Discussion

When the COVID-19 pandemic broke out, the effects of the virus on pregnancy and the fetus were unknown. Pregnant women were therefore among those told to avoid any unnecessary contact with others. Thus, instead of having to cope primarily with manageable changes, they were now faced with a less manageable and potentially more dangerous and tangible threat.

According to Modell [32,33], trauma “freezes” the past, preventing the formation of links with the present. Since traumatic memories of past events remain unprocessed, no meaningful connection to the present can be created. This, in turn, inhibits the possibility of imagining a future [29]. Pregnancy and motherhood involve the past, the present and the future. A woman must be in touch with her inner child and her internalized mother in order to be able to imagine her future and the future of her baby.

While there is no doubt that the first wave of the pandemic impeded the experience of continuity, replacing it with a sense of intrusion and ambiguity, the second wave had different characteristics. By this time, more information was available, and there was a degree of optimism regarding an imminent vaccine [31]. On the other hand, morbidity and mortality rates in the second wave were much higher, including among pregnant women, increasing the fear of giving birth in the shadow of COVID-19.

The two clinical cases above were chosen because they represent the response to the two waves of the pandemic of women who share similar characteristics: both were temperamental and anxious, had little self-compassion, were prone to pessimism, and perceived their social support as partial. At the same time, both were able to lean on and accept the therapist’s help. During the first wave, N. felt very anxious, whereas S. did not experience much anxiety during the second wave despite her similar lack of optimism. Furthermore, N. lacked confidence in the medical staff and felt they were unable to assist her properly during the pandemic, while S. had more confidence in the medical community and remained hopeful that a vaccine for the disease would be invented that would mitigate the danger.

As a therapist, I found treating N. during the first lockdown particularly difficult and complicated due to my own anxiety, uncertainty, and fear. In other words, I also needed time to process the situation and gain strength in order to be a container for N’s anxieties. When treating S., however, we were both able to engage in the typical concerns of pregnancy, focusing to a lesser degree on pandemic-related issues.

## 3. Part 2: Empirical Study

In light of previous findings [6], the quantitative study examined the impact on CCA in the first two waves of the pandemic of a variety of factors: sociodemographic characteristics, pregnancy-related variables, psychological distress, COVID-19-related concerns over economic damage, and the personal resources of optimism, self-compassion, and social support. In addition, it compared two subpopulations, Jews and Arabs. Jews represent the majority of the population in Israel, while Arabs constitute a meaningful minority (21%). As compared to their Jewish counterparts, Arab women report higher distress during the perinatal period [34,35], along with greater fear and concern for their own safety and health and that of the fetus during childbirth, both in general [36], and specifically during the pandemic [6].

In view of the literature, the following hypotheses were formulated.

**Hypothesis** **1.** *Negative associations will be found between pregnant women’s personal resources and their CCA: the higher their self-compassion, optimism, and perceived social support, the lower their anxiety will be*.

**Hypothesis** **2.** *Positive associations will be found between pregnant women’s psychological distress and COVID-19-related economic concerns and their CCA, so that the higher the distress and economic concerns, the higher the anxiety they will report*.

**Hypothesis** **3.** *Arab women will report higher CCA than Jewish women*.

In addition, given the lack of previous research on which to rely, the following questions were examined exploratively:

1. Is there a difference in CCA between Wave 1 and Wave 2 of the pandemic?
2. What is the unique and combined contribution of the study variables to CCA in the two waves?
3. Does wave of pandemic moderate the associations between the study variables and CCA? In other words, do the associations between the study variables and CCA be different according to the pandemic wave?

### 3.1. Method

#### 3.1.1. Participants and Procedure

This study was part of a larger research project that examined Israeli pregnant women’s psychological experience during the COVID-19 pandemic [5,6]. Consequently, certain aspects of the sample of women in Wave 1 of the current study were reported in a previous article [6].

After receiving approval from the university’s Institutional Review Board, a convenience sample of Jewish and Arab Israeli women was recruited for the study by means of a request posted on social media groups for women in general, and specifically for pregnant women, from 18 March to 9 April 2020. Participants were considered eligible for the study if they were pregnant and indicated that they could complete questionnaires in Hebrew. Of the 799 women who opened the questionnaire, 403 completed it in full (50.4% response rate): 233 Jewish and 170 Arab women. A second convenience sample of 1401 Israeli women, 593 Jewish and 808 Arab, was recruited in a similar manner from 5 July to 7 October 2020 (55% response rate), during the second wave of the pandemic. This time, both a Hebrew and Arabic version of the questionnaire was provided, and participants were considered eligible for the study if they were over the age of 18, pregnant, and indicated that they could complete questionnaires in Hebrew or Arabic. In both phases, the women were ensured the anonymity and confidentiality of the information, and were informed that they could cease to participate at any stage should they wish to do so. Moreover, they were told that they could call or email the researchers, whose contact details were supplied, if they felt any distress during or after completing the questionnaire.

A power analysis conducted with G*Power suggested we would need a sample size of 210 (105 in each group). We conservatively recruited above our desired sample size to account for attrition and strengthen the findings.

The final sample for both waves of the study thus consisted of 1804 pregnant women. For the sample as a whole, mother’s age ranged from 18 to 47 (M = 28.5, SD = 4.54), and gestational week from 4–42 (M = 27.6, SD = 9.83). Ninety-nine percent of the participants were married or in a spousal relationship; 76% had an academic degree, and the rest had a high school or post-high school diploma; 76.3% defined their income as average, 14.6% as above average, and 9.1% as below average; and 82.2% defined their health status as very good or good, 6.3% as average, and the rest (1.5%) as poor.

A series of t-tests and chi-square tests indicated that women in Wave 2 were slightly younger than those in Wave 1 (M = 28.19, SD = 4.39; M = 30.07, SD = 4.77, respectively), t(1757) = 7.47, *p* < 0.001, were in a slightly earlier gestational week (M = 24.36, SD = 9.92; M = 25.47, SD = 9.45, respectively), t(1796) = 1.99, *p* = 0.047, reported a somewhat lower economic status (above average, 12.4% vs. 22.2%, respectively), χ^2^ = 24.23, *p* < 0.001, and already had at least one child (63.2% vs. 55.6%, respectively), χ^2^ = 7.69, *p* < 0.01. Moreover, a lower proportion of participants in Wave 2 had undergone fertility treatment to achieve pregnancy (8.8% vs. 13.5%, respectively), χ^2^ = 15.98, *p* < 0.001, and a higher proportion had an academic degree (87.5% vs. 72.7%, respectively), χ^2^ = 41.21, *p* < 0.001. These differences were controlled for in the analysis.

#### 3.1.2. Instruments

**The Mental Health Inventory-Short Form** (MHI-5) [37] was used to assess psychological distress. Derived from the original MHI [38], the inventory is comprised of 5 items relating to the participant’s well-being and distress during the past week. Responses were indicated on a scale ranging from 1 (never) to 6 (all the time). Cronbach’s alpha in the current study was 0.82. An average score was therefore calculated, with a higher score reflecting greater psychological distress.

**The Self-Compassion Scale-Short Form** (SCS-SF) [39], which consists of 12 items representing a positive and negative indication of each of the three elements of self-compassion: self-kindness, common humanity, and mindfulness. Participant indicated their responses on a 7-point scale ranging from 1 (almost never) to 5 (almost always). In the current study, Cronbach’s alpha for the whole scale was 0.79. Each participant was assigned a single self-compassion score equal to the average of her responses to all items, with higher scores indicating a higher level of this resource.

**The Life Orientation Test** (LOT) [40], with only the four items relating to optimism employed in this study. Responses were indicated on a 5-point scale from 1 (strongly disagree) to 5 (strongly agree). Cronbach’s alpha for these items was 0.74, and thus a mean score was calculated, with higher scores indicating higher optimism.

**The Multidimensional Scale of Perceived Social Support** (MPSS) [41], which consists of 12 items relating to the perception of support from the family, friends, and a significant other. Responses were indicated on a 7-point scale from 1 (very strongly disagree) to 7 (very strongly agree). Cronbach’s alpha for the whole scale in the current study was 0.90. The average of the responses to all items served as the participant’s social support score, with higher scores indicating higher perceived support.

**COVID-19-related economic concerns** [5] was assessed by means of one item: “How anxious are you about the economic damage that may be caused to you and your family by the pandemic?” Responses were marked on a 5-point scale from 1 (very little) to 5 (very much), with higher scores indicating a higher level of COVID-19-related economic concerns.

**COVID-19-related childbirth anxiety** [6] was measured by a single item: “How anxious are you about delivery during the pandemic?” Participants marked their responses on a 5-point scale ranging from 1 (very little) to 5 (very much), with higher scores indicating a higher level of CCA.

**A sociodemographic questionnaire** was employed to obtain the background characteristics of the participants, specifically: age, education (1 = elementary; 2 = high school; 3 = post high school; 4 = academic), economic status (1 = below average; 2 = average; 3 = above average), physical health (1 = poor; 2 = average; 3 = good; 4 = very good), marital status (1 = single; 2 = married; 3 = in a couple relationship without marriage), ethnicity (0 = Jewish; 1 = Arab), gestational week, parity (0 = primiparous; 1 = multiparous), at-risk pregnancy (0 = diagnosis of a risk factor; 1 = not at risk), and whether or not the women had undergone fertility treatment to conceive (0 = spontaneous pregnancy; 1 = fertility treatment).

#### 3.1.3. Data Analysis

Analyses were conducted using SPSS (ver. 24). First, a series of t-tests were computed to examine differences in the study variables between Waves 1 and 2. We then calculated the correlations between the study and background variables and the level of CCA. Finally, a 5-step hierarchical regression was performed to determine the contribution of the independent variables to childbirth anxiety during the pandemic. The variables were entered as follows: Step 1, sociodemographic variables; Step 2, variables related to the pregnancy; Step 3, psychological distress and COVID-19-related concerns over economic damage; Step 4, the personal resources of optimism, self-compassion, and perceived social support; Step 5, interactions between wave and study variables. Analysis of the source of the interactions was performed using the PROCESS procedure [42].

### 3.2. Results

**Differences in the study variables in Waves 1 and 2.** The means and standard deviations of the study variables by wave, along with the results of the t-tests, appear in Table 1. As can be seen from the table, only CCA was higher during the first wave of the pandemic than during the second.

**Associations between study variables and CCA.**Table 2 presents the Pearson correlations between the study variables and CCA, indicating that higher level of education, poorer physical health, being an Arab woman, later gestational week, and at-risk pregnancy were all associated with higher CCA. In addition, higher psychological distress and economic concerns and lower perceived social support, optimism, and self-compassion were also significantly associated with higher CCA.

**Figure 1:**
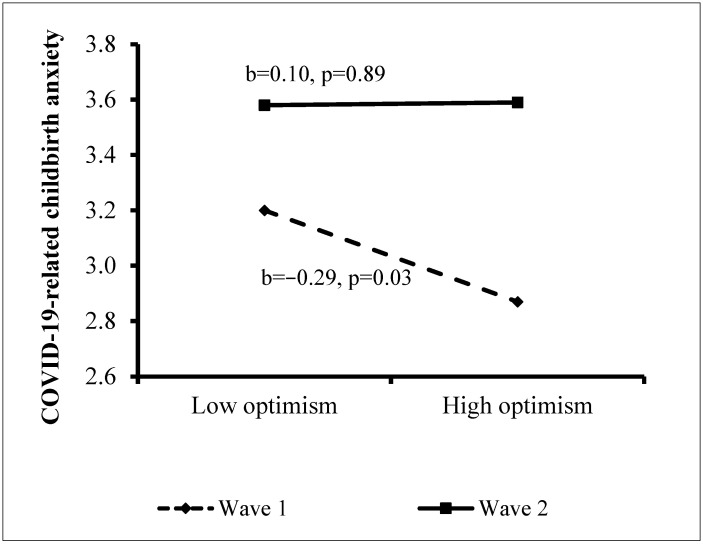
Effect of the interaction between wave and optimism on COVID-19-related childbirth anxiety.

**Contribution of the study variables to CCA.** The results of the regression analysis appear in Table 2. The independent variables explained 19.1% of the variance in CCA. Background characteristics in Step 1 contributed a significant 1.9% to the explained variance, with poorer health, higher level of education, and being an Arab woman contributing significantly to higher CCA. Step 2 added 2.6% to the explained variance, with later gestational week and at-risk pregnancy contributing significantly. Wave in Step 3 contributed 0.4% to the explanation of the variance, with CCA higher in Wave 1. In Step 4, higher psychological distress and economic concerns added a further 12.9% to the explained variance. The personal resources in Step 5 contributed an additional 0.8% to the explanation of the variance, with only self-compassion making a significant contribution, so that the lower the self-compassion, the higher the anxiety. Finally, the interactions in Step 6 added a further 0.5% to the explained variance, indicating a significant interaction between wave and optimism. Analysis of the source of the interaction (Hayes, 2017) revealed a negative association between optimism and CCA among women in Wave 1 (b = −0.29, *p* = 0.03), but this association was not significant in Wave 2 (b = 0.01, *p* = 0.89). That is, only during the first wave of the pandemic, was higher optimism associated with lower CCA (Figure 1).

## 4. General Discussion

The prolonged period of living under the threat of COVID-19 has impacted every conceivable domain of life. However, whereas the health and economic costs have been discussed from the beginning, the psychological implications, especially for vulnerable populations such as pregnant women, have only gradually attracted public and academic interest. This study compares the anxiety aroused by being pregnant during the first wave of the pandemic in Israel with the situation six months later, during the second wave. Moreover, the empirical study relates to two distinct ethnic groups, seeking to gain greater understanding of the differential implications of the pandemic for the CCA of Jewish and Arab pregnant women. In addition, the empirical data is joined by insights gained from two clinical case studies of pregnant women during the two waves of the pandemic.

A major finding of both the empirical and clinical studies is that CCA was lower in the second wave of the pandemic than in the first. This was found even after controlling for sociodemographic and pregnancy variables in the empirical study, and comparing women with similar personality traits in the clinical studies. Thus, it would appear that women adapted to a certain extent to the notion of giving birth during the crisis, a process that may have been aided by the greater information available and better organization of medical facilities over the course of time. To the best of our knowledge, no previous studies have been conducted on the CCA of pregnant women at these two points in time. However, studies conducted in the general population at different phases of the pandemic indicate fluctuations in the sense of danger and well-being in view of changes in external reality [17]. Our findings suggest that despite the greater intensity of the second wave of the pandemic, and the significant danger it posed, there may have been a better understanding of the virus, less ambiguity, and a certain adaptation to the prolonged crisis, leading to lower anxiety regarding childbirth in the shadow of COVID-19.

Another important finding concerns the background variables found to contribute to CCA. According to the empirical findings, the significant contributors to higher anxiety were higher level of education, poorer health, Arab ethnicity, later gestation week, and at-risk pregnancy. It may be assumed that women were more worried about the threat of the pandemic the closer their due date, particularly if their health or pregnancy was compromised [5]. In regard to education, a study of Irani pregnant women conducted at the beginning of the pandemic [43] found that higher level of education of the pregnant woman’s spouse predicted the woman’s stress, anxiety, and depressive symptoms. The authors suggest that higher level of education may facilitate greater understanding of the situation and the use of a wider array of information sources, thus leading to a more sensitive reaction to events.

The results of the empirical study also confirm Hypothesis 1, highlighting the important contribution of personal resources to CCA, with higher optimism, self-compassion, and perceived support from others all correlating with lower CCA. It is interesting to note that the results of the regression analysis indicated that after accounting for all other variables, only self-compassion was significantly associated with a lower level of anxiety. This finding provides further evidence that self-compassion can serve as a shield against anxiety in stressful situations [20]. The current study, however, is among the first to report an association between self-compassion and childbirth anxiety. This is particularly meaningful in the context of the pandemic, which aroused unusual stress and demanded social isolation. It suggests that when a woman’s has the ability to be forgiving, generous, and less judgmental toward herself, and to realize that she is part of a group that is sharing her experience, she may feel less anxious even in extremely stressful conditions.

In addition, a moderating role was found for study phase in the association between optimism and CCA, suggesting a distinction between the factors contributing to anxiety in the two waves of the pandemic. Thus, whereas lower optimism was related to higher CCA in Wave 1, no connection was found between optimism and anxiety in Wave 2. This finding is also confirmed by the clinical case studies. It is possible that lower optimism was associated with greater anxiety in the early days of the pandemic, when ambiguity was highest and there were considerable changes in the procedures surrounding childbirth. However, this resource, or lack thereof, may no longer have been relevant about six months later, when the situation was somewhat clearer and more information was available, despite the higher morbidity and mortality rates.

Finally, higher social support was found to be weakly related to lower CCA, and did not contribute to it in the presence of the background and psychological variables. The negative association between social support and anxiety is in line with the results of other studies conducted during the pandemic [8], and reflects the protective role such support plays in times of threat. However, the regression analysis indicated that other variables may have been more strongly related to women’s CCA, perhaps because the delivery would take place in a hospital, where she would be surrounded by healthcare professionals rather than by her social network. Pregnant women may therefore have been more concerned about the conduct of the medical staff during the crisis, or the effect of the pandemic on their experience of childbirth and their own safety. Moreover, Lebel et al. [8] found that higher symptoms of anxiety and depression among pregnant women during the pandemic were associated with greater concerns about the social isolation imposed, not receiving the expected prenatal care, and the threat to the health of the mother and baby. These concerns may have been accentuated by the fact that the woman was allowed to be accompanied to the hospital by only one individual, and was thus aware that she would not able to lean on her social network as she would in routine times. Consequently, social support may have served as a less meaningful asset in the context of childbirth anxiety during the pandemic than it would have otherwise, as evidenced by the clinical cases as well.

The empirical findings also confirmed Hypothesis 2, showing that greater psychological distress and economic concerns were both associated with higher CCA. This is in line with previous studies [14], and suggests that CCA may be part of a more generalized poorer psychological profile. In addition, in confirmation of Hypothesis 3, Arab women reported higher levels of CCA than their Jewish counterparts. This is consistent with the finding in the general population that Israeli Arabs reported higher distress during the pandemic [44], as well as with studies indicating an association between ethnicity and general FOC [14]. The explanation for the greater vulnerability of Arab women may lie in the fact that, on the whole, Arab society in Israel suffers from more crowded living conditions, lower income per family, and poorer access to health services as compared to Jewish society [45]. In other words, the social gaps between the two communities may also be reflected in the level of anxiety aroused by childbirth in crisis conditions.

Certain limitations of the current study should be noted. First, the sample in the empirical study was not representative. Secondly, the proportion of Arab women in the sample was higher than their proportion in the Israeli population. This was done in order to enable a comparison between Jewish and Arab women. Moreover, we wished to identify the factors that contribute to CCA in the two communities, regardless of proportions. Finally, the proportion of women with an academic education in the sample was higher than in the general population. This is often the case in non-representative samples, since women with more education tend to participate more in research studies [46].

These limitations notwithstanding, we believe the study makes an important and novel contribution to the literature. One of its major strengths is that it relies on both empirical and clinical evidence collected in different phases of the ongoing pandemic. Future studies conducted among pregnant women at additional points in time during the crisis may be able to increase our understanding of its effect on their anxieties, as well as the implications for both mother and child. In addition, the study relates not only to the vulnerable population of pregnant women in general, but also to two subpopulations with different religions and cultures. The results suggest that for Arab women, the double vulnerability of being pregnant and belonging to an ethnic minority may pose a greater risk of childbirth anxiety during a crisis. This is an important insight for healthcare professionals.

The findings also highlight the importance of self-compassion in reducing childbirth anxiety, suggesting that professionals should attempt to strengthen this personal resource. In a period of social isolation and health threats, women’s ability to care for themselves in an enduring and emphatic way may be critical to their psychological health and coping ability. Given that the crisis is not yet over, all efforts should be made to ensure the health of pregnant women, both physical and psychological, as they are at even higher risk than usual of perinatal and postpartum depression, as well as other psychopathological disorders.

Clinical implications evolving from this study involve providing information to pregnant women about pregnancy during a pandemic in community clinics, such as women’s health clinics that combine mental and medical treatments, and ensuring follow-ups during pregnancy. Less ambiguity of the entire health system concerning the characteristics of the pandemic would help to gain better adaptation of women and lead to lower anxiety regarding childbirth in the shadow of the medical threats. It should be noted that most of the Arab population lives in the periphery of Israel, where some medical services are less accessible. This may impair their ability to receive regular or emergency medical care [45] in general, and in times of crisis such as COVID-19, in particular. Mental health nurses, social workers and psychologists in the community should help locate women who suffer from higher anxieties, reach out to, and help them find community support and family support systems, and strengthen their inner resources in such times of a global crisis. Interventions should be culturally sensitive and take into account cultural and other differences (such as women who live in the center of the country vs. those from the periphery) between women, to reach effective intervention outcomes.

## 5. Conclusions

The study brings together empirical and clinical evidence from two phases of the COVID-19 pandemic, highlighting the similarities and differences between these phases. Moreover, the study relates to two subpopulations of pregnant women with different backgrounds in regard to religion and culture. The results suggest that for Israeli Arab women, the double vulnerability of being pregnant and belonging to an ethnic minority may pose a greater risk of childbirth anxiety during a crisis. The findings also highlight the importance of protective factors, such as self-compassion, in reducing childbirth anxiety. It highlights the fact that in a period of social isolation and health threats, women’s ability to care for themselves in an emphatic mode may be critical to their psychological health and coping ability.

## Figures and Tables

**Table 1:** Means, Standard Deviations, and t-tests for the Study Variables by Wave.

	Wave 1 (*n* = 1375)		Wave 2 (*n* = 402)			
	M	SD	M	SD	Cohen’s d	t
Psychological distress	3.05	0.88	3.05	0.91	0.01	−0.13
COVID-19-related economic concerns	3.24	1.10	3.20	1.15	0.04	0.65
Optimism	2.62	0.59	2.58	0.57	0.07	1.24
Self-compassion	3.28	0.63	3.49	0.63	0.10	−1.79
Social support	5.83	0.93	5.81	0.99	0.02	0.37
COVID-19-related childbirth anxiety	3.59	1.18	3.45	1.14	0.12	2.10 *

* *p* < 0.05.

**Table 2:** Pearson Correlations and Hierarchical Regression Coefficients (Beta Weights) for COVID-19-related Childbirth Anxiety.

	COVID-19-Related Childbirth Anxiety			
	r	ß	t	∆R^2^
**Step 1**				0.019 ***
Age	−0.02	−0.03	−1.11	
Education	0.05 *	0.05	1.97 *	
Economic status	−0.02	−0.02	−0.61	
Physical health	−0.11 ***	−0.11	−4.28 ***	
Ethnic group ^a^	0.08 ***	0.05	1.99 *	
**Step 2**				0.026 ***
Gestation week	0.12 ***	0.14	5.35 ***	
Parity ^b^	−0.10	−0.01	−0.29	
At-risk pregnancy ^c^	0.08 ***	0.09	3.27 **	
Fertility treatments ^d^	−0.02	−0.03	−1.10	
**Step 3**				0.004 *
Wave ^e^	−0.05 *	-0.06	−2.41 **	
**Step 4**				0.129 ***
Psychological distress	0.31 ***	0.25	9.49 ***	
COVID-19-related economic concerns	0.30 ***	0.24	9.69 ***	
**Step 5**				0.008 **
Optimism	−0.19 ***	−0.03	−0.09	
Self-compassion	−0.18 ***	−0.08	−2.85 **	
Social support	−0.07 **	0.02	0.86	
**Step 6**				0.005 *
Wave × Psychological distress		−0.13	−1.10	
Wave × COVID-19-related economic concerns		−0.03	−0.30	
Wave × Optimism		0.31	2.05 *	
Wave × Self-compassion		−0.21	−1.23	
Wave × Social support		0.09	0.52	
R^2^				19.1
*F* (20, 1472)				17.37 ***

^a^ 0 = Jewish women, 1 = Arab women; ^b^ 0 = primiparous, 1 = multiparous; ^c^ 0 = No, 1 = Yes; ^d^ 0 = No, 1 = Yes; ^e^ 0 = Wave 1, 1 = Wave 2. * *p* < 0.05, ** *p* < 0.01, *** *p* < 0.001.

## Data Availability

The data that support the findings of this study are available from the corresponding author upon reasonable request.
